# Angiopoietin-1 Mimetic Nanoparticles for Restoring the Function of Endothelial Cells as Potential Therapeutic for Glaucoma

**DOI:** 10.3390/ph15010018

**Published:** 2021-12-24

**Authors:** Raphael Mietzner, Ramona Pawlak, Ernst R. Tamm, Achim Goepferich, Rudolf Fuchshofer, Miriam Breunig

**Affiliations:** 1 Department of Pharmaceutical Technology, University of Regensburg, Universitaetsstrasse 31, 93040 Regensburg, Germany; raphael.mietzner@chemie.uni-regensburg.de (R.M.); Achim.Goepferich@chemie.uni-regensburg.de (A.G.); 2 Department of Human Anatomy and Embryology, University of Regensburg, Universitaetsstrasse 31, 93040 Regensburg, Germany; Ramona.Pawlak@ur.de (R.P.); Ernst.Tamm@ur.de (E.R.T.); Rudolf.Fuchshofer@ur.de (R.F.)

**Keywords:** glaucoma, POAG, Schlemm’s canal, Tie2, angiopoietin 1, Angpt-1 mimetic, eNOS, nanoparticles, peptide

## Abstract

A root cause for the development and progression of primary open-angle glaucoma might be the loss of the Schlemm’s canal (SC) cell function due to an impaired Angiopoietin-1 (Angpt-1)/Tie2 signaling. Current therapeutic options fail to restore the SC cell function. We propose Angpt-1 mimetic nanoparticles (NPs) that are intended to bind in a multivalent manner to the Tie2 receptor for successful receptor activation. To this end, an Angpt-1 mimetic peptide was coupled to a poly(ethylene glycol)-poly(lactic acid) (PEG-PLA) block co-polymer. The modified polymer allowed for the fabrication of Angpt-1 mimetic NPs with a narrow size distribution (polydispersity index < 0.2) and the size of the NPs ranging from about 120 nm (100% ligand density) to about 100 nm (5% ligand density). NP interaction with endothelial cells (HUVECs, EA.hy926) as surrogate for SC cells and fibroblasts as control was investigated by flow cytometry and confocal microscopy. The NP–cell interaction strongly depended on the ligand density and size of NPs. The cellular response to the NPs was investigated by a Ca^2+^ mobilization assay as well as by a real-time RT-PCR and Western blot analysis of endothelial nitric oxide synthase (eNOS). NPs with a ligand density of 25% opposed VEGF-induced Ca^2+^ influx in HUVECs significantly which could possibly increase cell relaxation and thus aqueous humor drainage, whereas the expression and synthesis of eNOS was not significantly altered. Therefore, we suggest Angpt-1 mimetic NPs as a first step towards a causative therapy to recover the loss of SC cell function during glaucoma.

## 1. Introduction

Primary open-angle glaucoma (POAG) is a chronic, progressive neuropathy of the optic nerve and one of the leading causes of blindness worldwide [1,2]. Intraocular pressure (IOP) is considered as the only modifiable risk factor for POAG development and progression [3]. The IOP is generated in the anterior chamber of the eye and is maintained by the balance between the production of aqueous humor in the ciliary body and its efflux through the trabecular outflow pathway. Pathologically altered tissues in the trabecular outflow system that are accountable for an increased IOP are the juxtacanalicular connective tissue (JCT), together with the inner wall endothelium of the Schlemm´s canal (SC) [4,5].

Most anti-glaucoma drugs on the market act only symptomatically and do not engage the pathological changes in the trabecular outflow system. More innovative drugs that were recently approved, are Rho kinase inhibitors such as netarsudil (Rhopressa) and NO donors such as latanoprostene bunod (VYZULTA). They address the abnormally higher extracellular matrix (ECM) synthesis and increased cell contractility of JCT and SC cells. However, they still fail to rescue the function of the SC [2,5,6,7]. As yet, there is no drug on the market that specifically targets the inner wall endothelium of the SC. A certain level of SC cell loss has a more prominent negative impact on IOP compared to the aforementioned ECM accumulation in the JCT [8]. Therefore, it is of utmost importance to specifically target SC cells, especially after disease has progressed.

**Scheme 1:**
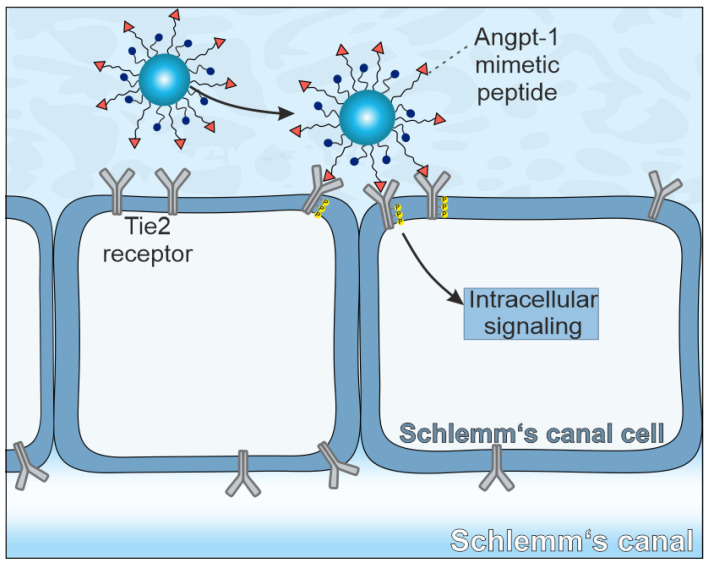
Concept of multivalent Angpiopoietin-1 (Angpt-1) mimetic nanoparticles (NPs) intended to restore Schlemm’s canal (SC) function. Polymer NPs are surface functionalized with the Angpt-1 mimetic peptide sequence HHHRHSF. After reaching SC cells, particles bind to the Tie2 receptor in a multivalent manner, inducing Tie2 receptor clustering and activation. After activation of the signaling cascade, several signaling pathways (not shown) are activated, restoring SC cell function. The dimensions do not correspond to reality.

Recently it was demonstrated that the integrity and functionality of SC is maintained by the signaling between angiopoietin 1 (Angpt-1) and its tyrosine kinase receptor Tie2 [9]. Reduction or even inactivation of the Angpt-1/Tie2 signaling during adulthood induces SC degeneration and is a key factor for IOP disbalance [4,9,10]. Restoring this pathway by adding recombinant Angpt-1 as a therapeutic agent is not straightforward because Angpt-1 is prone to aggregation and is therefore not suitable as a therapeutic agent [11]. Recently, an Angpt-1 mimetic peptide sequence (HHHRHSF) was discovered [12,13,14]. We hypothesize, that the Angpt-1 mimetic peptide immobilized on the surface of nanoparticles (NPs) could be able to restore SC cell function. For successful receptor activation, the clustering of multiple Tie2 receptors is required [15]. Because NPs may bind to several cell surface receptors simultaneously in a so-called multivalent manner, the crosslinking of multiple Tie2 receptors should thus be possible (Scheme 1).

In the present study, our aim was to take a first step towards developing a first therapeutic targeting the Tie2 receptor. Therefore, we developed Angpt-1 mimetic NPs. For this purpose, we attached the Angpt-1 mimetic peptide covalently to a poly(ethylene glycol)—poly(lactic acid) (PEG-PLA) block copolymer. In the next step, we used the modified polymer together with the polymer poly(lactic-co-glycolic acid) (PLGA) to produce NPs. First, we characterized the NPs physicochemically. For cellular experiments, three different cell types including a human endothelial cell line (EA.hy926) as surrogate cells for SC cells, human umbilical vein endothelial cells (HUVECs) as the primary counterpart and primary fibroblasts as control cells were chosen. All three cell types were examined regarding their Tie2 receptor expression levels. Subsequently, NP uptake experiments were performed, and the cellular effects were demonstrated by a Ca^2+^ mobilization assay and real-time reverse transcriptase (RT)-PCR and Western blot analyses of endothelial nitric oxide synthase (eNOS).

## 2. Results

### 2.1. Preparation and Characterization of Multivalent Angpt-1 Mimetic NPs

For the development of Angpt-1 mimetic NPs, we chose polymer NPs on the basis of PEG-PLA block co-polymers and biodegradable PLGA, both known for their excellent biocompatibility (Figure S1) [16,17,18,19]. Such a particle system enables the premodification of block co-polymers with a peptide sequence allowing for precise control of the NP composition and thus offering the possibility of modular NP preparation [20,21].

**Figure 1:**
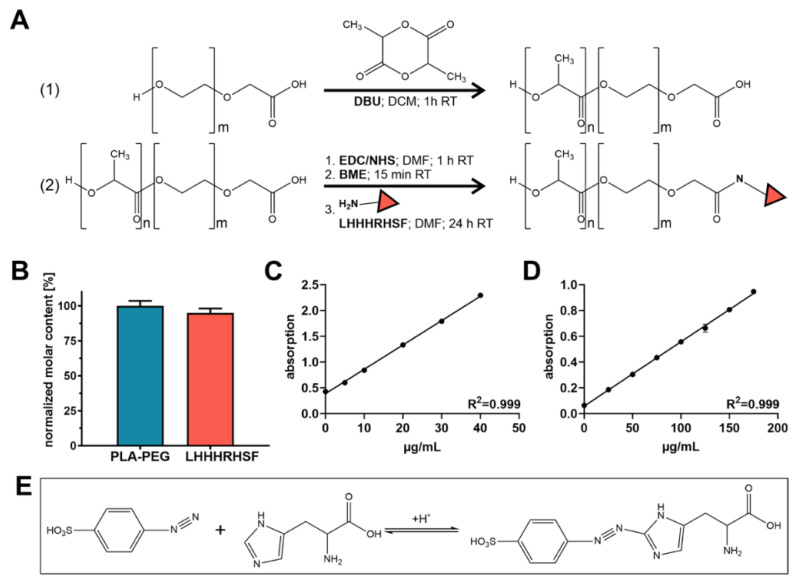
Polymer synthesis and characterization. (**A**) Exemplary synthesis procedure for peptide-modified PEG_5k_-PLA_10k_ block co-polymer. (1) The PLA block was coupled via ring-opening polymerization of 3,6-dimethyl-1,4-dioxane-2,5-dione to hetero-bifunctional PEG polymer (m: PEG block of 5 kDa length; *n*: PLA block of 10 kDa length). (2) In a next step, COOH-PEG_5k_-PLA_10k_ was attached to the leucine residue of HHHRHSF via EDC/NHS (1-ethyl-3-(3-dimethylaminopropyl)carbodiimide/N-hydroxysuccinimide) chemistry (for more details, please refer to Section 4). (**B**) Coupling efficiency of synthesized PLA_10k_-PEG_5k_-LHHHRHSF was determined by independently measuring the concentration of both PEG and LHHHRHSF using the iodine-assay ((**C**) corresponding calibration curve) and the Pauly reaction ((**D**) corresponding calibration curve), respectively. The coupling efficiency between polymer and peptide was about 95%. (**E**) Principle of the Pauly reaction that specifically detects histidine. Diazotized sulfanilic acid forms under alkaline conditions with histidine the red to orange colored c-azo complex. Absorbance of the solution was measured at λ = 490 nm. Results are presented as mean ± SD of at least *n* = 3 measurements. DBU: 1,8-diazabicyclo [5.4.0] undec-7-ene; DCM: methylene chloride; BME: β-mercaptoethanol; DMF: dimethylformamide; RT: room temperature.

First, PEG-PLA polymers with either longer (COOH-PEG_5k_-PLA_10k_) or shorter (MeO-PEG_2k_-PLA_10k_) PEG chains were synthesized via ring-opening polymerization of cyclic lactide (Figure 1A). The combination of polymers of different lengths and terminal functionalization offered the possibility, later, to influence the particle size and to design NPs of varying ligand content per particle. Therefore, in a second step, HHHRHSF containing a leucine residue at the NH_2_ terminus was covalently coupled to the longer COOH-PEG_5k_-PLA_10k_ block copolymer via EDC/NHS 1-ethyl-3-(3-dimethylaminopropyl)carbodiimide/N-hydroxysuccinimide) activation. The coupling efficiency was around 95% as determined by an iodine assay and Pauly assay (Figure 1B–E). The shorter polymer (MeO-PEG_2k_-PLA_10k_) was not modified further.

**Figure 2:**
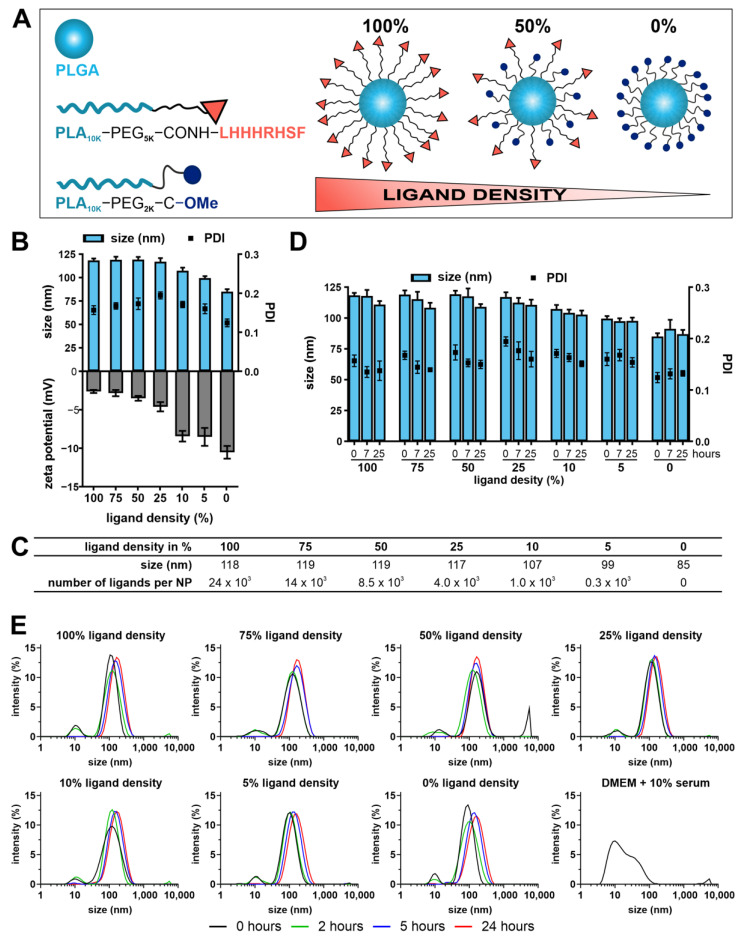
Preparation and characterization of Angpt-1 mimetic NPs. (**A**) Schematic illustration of particle compounds and composition. Longer and LHHHRHSF-functionalized PEG_5k_-PLA_10k_ polymer was mixed with shorter unfunctionalized MeO-PEG_2k_-PLA_10k_ in desired ratios as well as poly(lactic-co-glycolic acid) (PLGA) to manufacture multivalent Angpt-1 mimetic NPs of various surface ligand densities (100% to 0%). (**B**) Size determination by dynamic light scattering (DLS). NP size ranged from 120 to 90 nm with decreasing ligand density. The polydispersity index (PDI) was below 0.2 for each sample indicating no remarkable aggregation. Results are presented as mean ± SD of at least *n* = 3 measurements. (**C**) Number of ligands per NP. Shown are the absolute ligand numbers per NP corresponding to the percent ligand density. Values were calculated according to Abstiens et al. [20]. With decreasing ligand density, the size as well as the calculated number of ligands per particle decrease. (**D**) NP stability measurement in DPBS over 25 h. Particles were analyzed regarding size and PDI after 0, 7 and 25 h. Particles showed an overall stable particle size distribution over this time. Results are presented as mean ± SD of at least *n* = 3 measurements. (**E**) NP stability measurement in culture medium. Intensity-weighted average size distribution of NPs incubated in culture medium supplemented with 10% serum. Samples were analyzed after 0, 2, 5 and 24 h. Over the time, particles underwent a slight right shift.

Angpt-1 mimetic NPs are intended to specifically bind to Tie2 receptors in a multivalent manner. To guarantee that the Angpt-1 mimetic ligand on the particle surface is accessible for cells, the longer functionalized polymer was combined with the non-functionalized shorter polymer prior to NP preparation. Longer and shorter polymers and PLGA as the NP core stabilizing component were mixed in the desired ratios and NPs were prepared by bulk nanoprecipitation in 0.1 × DPBS. Thus, it was possible to produce NPs with a ligand density ranging from 100 to 0% (Figure 2A). With decreasing surface ligand density (100 to 0%), the NP size decreased from about 120 to about 90 nm (Figure 2B). The poly dispersity index (PDI) of all samples was below 0.2, indicating an overall monodisperse particle distribution without considerable aggregates. The zeta potential decreased from -3 to -10 mV in the same order (Figure 2B). Figure 2C shows the numbers of ligands per NP considering a spherical shape for the NPs (calculated according to Abstiens et al. [20]). With decreasing ligand density, the number of ligands per NP decreased from about 2.4 × 10^4^ (100% ligand density) over 8.5 × 10^3^ (50% ligand density) to about 3.4 × 10^2^ (5% ligand density). The particles were stable over 25 h in DPBS (37 °C) (Figure 2D). Only a slight decrease of the size as well as of the PDI was observed over this time period. To guarantee colloidal stability in cell experiments, we further investigated the particle size distribution in culture medium supplemented with 10% serum over 24 h (Figure 2E). A slight right shift of the main NP peak at around 100 nm was observed in all samples over the time. Additionally, the intensity of the smaller peak at around 10 nm which is associated with the presence of free serum protein such as albumin, decreased. These observations are indicative of serum protein adsorption to the NP surface [22].

### 2.2. Investigation of NP Interaction with Cells of Varying Tie2 Receptor Expression

A prerequisite for the specific interaction of Angpt-1 mimetic NPs with cells is a sufficiently high expression of the Tie2 receptor. For this reason, three different cell types were studied, regarding their Tie2 receptor expression. As the availability of SC cells is limited, and most important because SC cells usually lose essential signaling in conventional culture systems, HUVECs and EA.hy926 cells were used as surrogate for SC cells [23]. Using primary low-passage HUVECs instead of SC cells is a common procedure in literature [24]. The EA.hy926 cell line stably expresses Tie2 receptor over many passages [13,25]. Primary fibroblasts were used as control cells.

**Figure 3:**
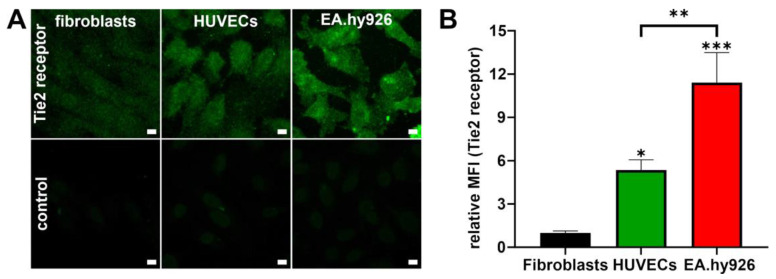
Comparison of Tie2 receptor expression levels. EA.hy926 cells, HUVECs and fibroblasts were analyzed regarding their expression level of the Tie2 receptor. (**A**) Fluorescence immunocytochemistry. Upper panels: Representative images of cell-associated Tie2 receptor fluorescence (green, lower panel). After permeabilization, cells were incubated with a primary anti-human Tie2 antibody followed by appropriate Alexa Fluor 488-labeled secondary antibody. EA.hy926 cells showed the highest fluorescence intensity, followed by HUVECs and fibroblasts. Lower panels: Staining without primary antibody showed a negligible background fluorescence intensity. Bars indicate 10 µm. (**B**) Flow cytometry analysis of the Tie2 receptor expression levels. Presented is the fold change of the cell-associated mean fluorescence intensity (MFI) of Tie2 receptor compared to fibroblasts. Again, after trypsinization, fixation and permeabilization, cells were incubated with a primary anti-human Tie2 antibody followed by appropriate Alexa Fluor 488-labeled secondary antibody. Tie2 receptor-derived fluorescence was excited at 488 nm and the emission was detected using a 660/20 nm bandpass filter. EA.hy926 cells showed a 11-fold higher and HUVECs a 5-fold higher MFI compared to fibroblasts. Results are presented as mean ± SD of at least *n* = 3 measurements. * *p* < 0.05, ** *p* < 0.01, *** *p* < 0.001.

Figure 3 shows the cellular Tie2 receptor expression level of these cell types. Initially cells were observed by confocal microscopy using a specific, primary anti-human Tie2 antibody followed by a fluorescently labeled secondary antibody (Figure 3A). A high fluorescence intensity of the Tie2 receptor was detected for EA.hy926, a less strong one for HUVECs and almost no fluorescence for fibroblasts. The first impression was confirmed by flow cytometric analysis (Figure 3B). Again, trypsinized and fixed cells were incubated with the primary antibody against Tie2 and the appropriate fluorescently labeled secondary antibody. The cell-associated Tie2 receptor mean fluorescence intensity (MFI) was measured for each cell type. For EA.hy926 cells the fluorescence intensity was significantly higher by 11-fold compared to fibroblasts, and for HUVECs by 5-fold, respectively.

**Figure 4:**
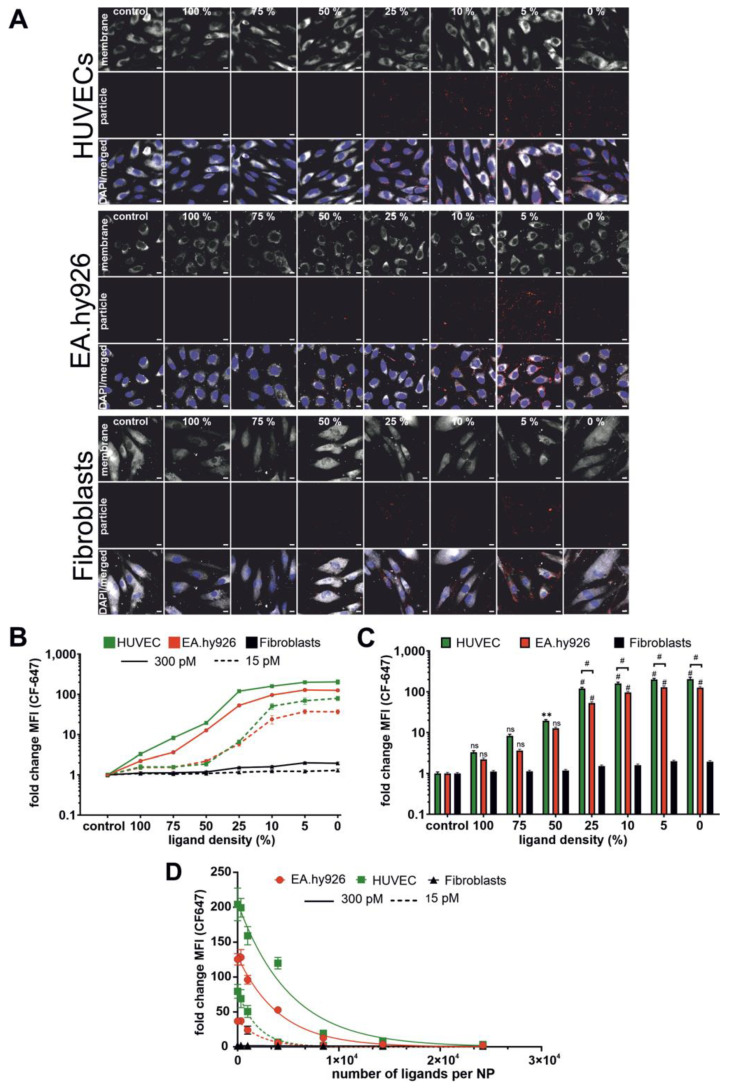
Interaction of Angpt-1 mimetic NPs with HUVECs, EA.hy926 cells and fibroblasts. (**A**) Representative fluorescence microscopy images of cells that were incubated with NPs (300 pM) for 2 h. NPs were labeled with CF 647 and are shown in red. The ligand density ranged from 100 to 0%. Plasma membrane was stained with CellMask and is shown in white. After incubation with NPs, cells were washed with DPBS, fixed and nuclei were stained with 4′,6-diamidino-2-phenylindole (DAPI) and are shown in blue. With decreasing ligand density, increasing levels of NP-associated fluorescence were detected. (**B**) Corresponding flow cytometry analysis of NP–cell interaction. Cells were incubated with NPs at a concentration of either 300 or 15 pM. Presented is the fold change of cell-associated mean fluorescence intensity (MFI) of NPs compared to untreated control cells. NPs were again labeled with CF 647. Angpt-1 mimetic NPs showed a substantially increased cell interaction with decreasing ligand density. The interaction was higher for HUVECs and EA.hy926 cells than for fibroblasts. (**C**) Statistical analysis of cells treated with a NP concentration of 300 pM. (**D**) Correlation between NP–cell interaction and number of ligands per NP. NP–cell interaction of HUVECs and EA.hy926 cells correlated with the number of ligands in a kind of exponential manner. Data were fitted using a one phase exponential equation model. Results are presented as mean ± SD of at least *n* = 3 measurements. ** *p* < 0.01, # *p* < 0.0001, ns: not significant.

To get a first impression of the NP–cell interaction, HUVECs, EA.hy926 cells and fibroblasts were observed by confocal microscopy after incubation with fluorescently labeled Angpt-1 mimetic NPs (Figure 4A). The surface ligand density of the NPs varied again from 100 to 0%. After incubation with NPs of the highest ligand content, almost no particle fluorescence was detected in all three cell types. With decreasing ligand density, more and more fluorescent spots were observed. These observations were consistent over all three cell types but were particularly pronounced for HUVECs and EA.hy926 cells and to a much lesser extent for fibroblasts.

To quantify the NP–cell interaction and to confirm the first visual impression, we performed in a next step a flow cytometry analysis. Cells were incubated with NPs at a concentration of either 300 or 15 pM for 2 h. Figure 4B,C show the fold change of mean NP fluorescence in comparison to untreated cells at each ligand density. In the case of HUVECs and EA.hy926 cells, fluorescence increased with decreasing ligand density, independent of the particle concentration. In general, the NP–cell interaction decreased in the following order: HUVECs > EA.hy926 > fibroblasts. A closer look at the data of the cells treated with 300 pM NP revealed that between 25 to 0% ligand density, the fluorescence was significantly (*p* < 0.0001) higher for both cell types compared to fibroblasts (Figure 4C). Plotting the fluorescence intensity against the ligand density demonstrated that the NP–cell interaction of HUVECs and EA.hy926 cells correlated with the number of ligands in a kind of exponential manner (Figure 4D).

### 2.3. Impact of Angpt-1 Mimetic NPs on VEGF-Induced Ca^2+^ Influx in Cells

**Figure 5:**
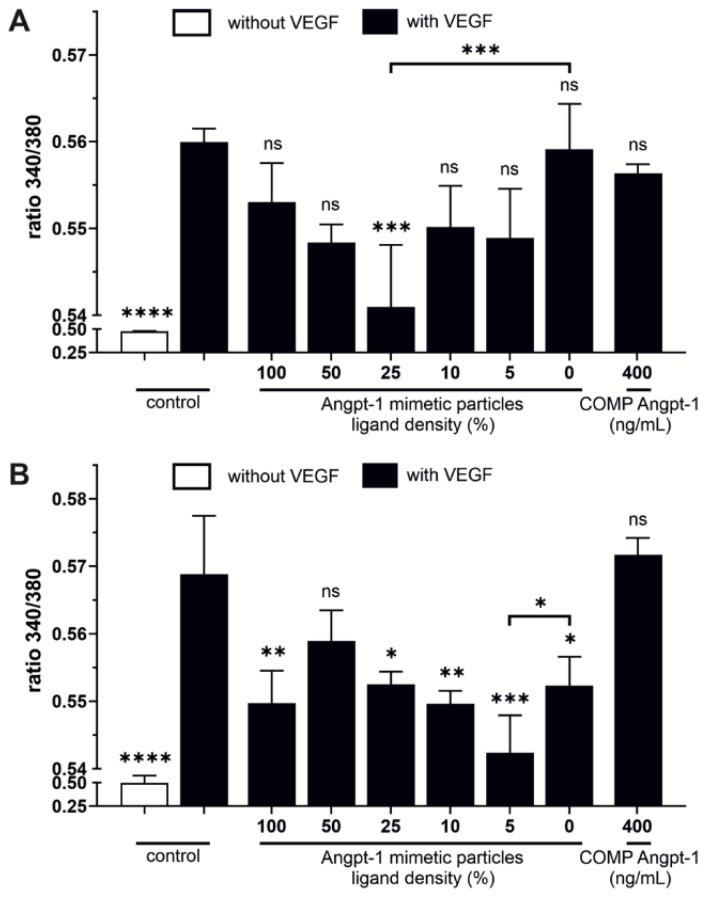
Angpt-1 mimetic NPs reduced VEGF-induced Ca^2+^ influx in endothelial cells. Cells were loaded with fura-2 AM for 2 h. Fura-2 AM allows for precisely measuring the intracellular concentration of Ca^2+^. After cell loading, HUVECs (**A**) and EA.hy926 cells (**B**) were incubated with Angpt-1 mimetic NPs (150 pM) of varying ligand density (100% to 0%) or COMP Angpt-1 at a concentration of 400 ng/mL, both in presence of VEGF. A concentration of 400 ng/mL for COMP Angpt-1 was chosen because it is in the range of concentrations usually used in the literature [11,27,28,29]. Presented is the Ca^2+^-dependent ratio of fluorescence emission of fura-2 AM at 510 nm after excitation at 330 and 380 nm, respectively. Angpt-1 mimetic particles opposed VEGF-induced Ca^2+^ influx in both HUVECs and EA.hy926 cells. Results are presented as mean ± SD of at least *n* = 3 measurements. * *p* < 0.05, ** *p* < 0.01, *** *p* < 0.001, **** *p* < 0.0001 ns: not significant. *Y*-axes are discontinued between 0.50 and 0.54 (**A**,**B**).

To investigate the cellular effects that were elicited by Angpt-1 mimetic particles, a Ca^2+^ mobilization assay was performed. From the literature, it is known that Angpt-1 inhibits extracellular Ca^2+^ influx and that vascular endothelial growth factor (VEGF) acts as its counterpart [26,27]. Intracellular Ca^2+^ levels were determined after loading cells with fura-2 AM and recording the fura-2 fluorescence emission after excitation at 340 and 380 nm. The higher the fluorescence emission ratio of fura-2 AM in Figure 5, the higher was the intracellular Ca^2+^ concentration and vice versa.

Cells were incubated with NPs of varying ligand density (100 to 0%) or with recombinant COMP Angpt-1, each sample in the presence of VEGF. Figure 5A shows the fluorescence emission ratio of HUVECs after 10 min of incubation. As expected, after incubation with VEGF only, the fluorescence emission ratio increased from a control level of 0.46 to a value of 0.56 which can be interpreted as an increase of the cellular Ca^2+^ level. Angpt-1 mimetic NPs seemed to attenuate this increase because the fluorescence emission ratio was lower than after application of VEGF alone. When NPs with a ligand density of 25% were applied, the effect was statistically significant (*p* < 0.01). The NPs of all applied ligand densities decreased the fluorescence emission ratio. Unexpectedly, incubation with COMP Angpt-1 at a concentration of 400 ng/mL led to a similar value as after incubation with VEGF. This means that soluble COMP Angpt-1 did not oppose the Ca^2+^ influx induced by VEGF to such an extent as the Angpt-1 mimetic NPs. EA.hy926 cells showed a similar trend with the exception that particles without ligand exerted an effect as well.

### 2.4. Impact of Angpt-1 Mimetic NPs on eNOS Expression

**Figure 6:**
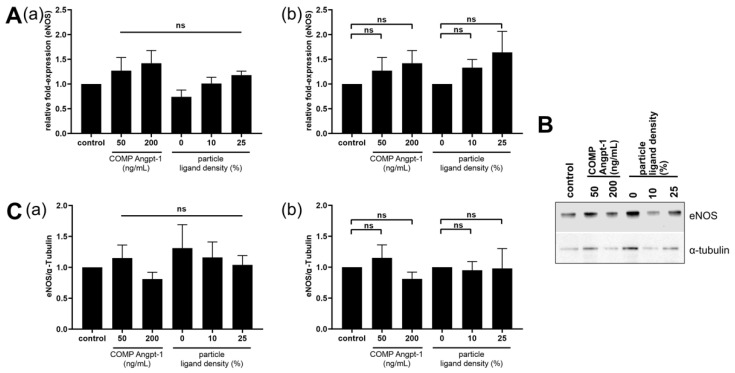
Impact of Angpt-1 mimetic NPs as well as COMP Angpt-1 on eNOS expression. HUVECs were incubated with Angpt-1 mimetic NPs (150 pM) of varying ligand density (0%, 10% and 25%) or COMP Angpt-1 (50 and 200 ng/mL) for 24 h. (**A**) Real time RT-PCR analysis of eNOS. Presented are the relative fold-expression levels of eNOS. (a) With increasing concentration of COMP Angpt-1, the expression of eNOS was 1.3-fold after 50ng/mL and 1.4-fold higher after 100 ng/mL relative to control. (b) Considering the Angpt-1 mimetic NP treated samples relative to particle control (NPs with 0% ligand density), the expression of eNOS was 1.3-fold and 1.6-fold higher with 10 and 25% ligand density, respectively. (**B**) Representative Western blots for eNOS. (**C**) Western blot analysis of eNOS. Presented are the relative ratios eNOS and α-tubulin expression. (a) Treatment with Angpt-1 mimetic NPs as well as COMP Angpt-1 has no impact on total eNOS expression. (b) Considering the expression ratios of Angpt-1 mimetic NP treated samples relative to 0% NP ligand density, no increase of total eNOS can be observed. Results are presented as mean ± SEM. ns: not significant.

To investigate the effect of Angpt-1 mimetic NPs on eNOS expression, a potential downstream target of the Tie2 signaling cascade [30], a real-time RT-PCR as well as a Western blot analysis was performed after cells were incubated for 24 h with COMP Angpt-1 (50 and 200 ng/mL) and Angpt-1 mimetic NPs of varying ligand densities (0–25% ligand density). Figure 6A shows the semi quantitative analysis of eNOS mRNA. With an increasing concentration of COMP Angpt-1, the expression of eNOS was 1.3-fold higher after 50ng/mL and 1.4-fold higher after 100 ng/mL relative to control. The same was true for the treatment with Angpt-1 mimetic NPs, as with a ligand density of 10% the eNOS expression was 1.3-fold and with 25% 1.6-fold higher compared to control NPs (0% ligand density). However, for both (NPs and COMP Angpt-1), the increase was not significant, which was confirmed on the protein level, where no increase of eNOS was observed after NP or protein treatment (Figure 6B,C).

## 3. Discussion

POAG is one of the leading causes of blindness worldwide. Due to its multifactorial disease character, the precise pathogenesis remains unclear [31]. However, in the last few years, therapies targeting the cells of the conventional outflow pathway to restore pathological changes and thereby decreasing IOP have become the focus of attention [3]. With the advent of rho-associated protein kinase (ROCK) inhibitors, the first drug class has been introduced for causative POAG management. However, market-ready approaches fail to counteract the endothelial cell loss that is associated with SC regression. With SC degeneration, important functions, such as transcellular aqueous humor filtration that are essential for IOP control, are lost [9]. To deal with these shortcomings, we propose in this study polymeric Angpt-1 mimetic NPs that specifically bind to the Tie2 receptor and restore SC cell function in the long run.

The Angpt-1 mimetic peptide sequence HHHRHSF has been applied in another context. Van Slyke et al. and David et al. both coupled the peptide sequence HHHRHSF to a branched PEG polymer for the treatment of diabetic ulcera and microvascular endothelial barrier dysfunction, respectively [12,13,14]. Thus, it was possible to couple a maximum of four peptide molecules per PEG molecule. In contrast, we coupled the peptide to a linear PEG-PLA block co-polymer. Thus, on the one hand only about one molecule per polymer chain was attached. On the other hand, our strategy allows for the formation of NPs. Consequently, a much higher number of ligands per particle was possible. To give an impression: at a ligand density of 100%, one NP carries about 24 × 10^3^ ligands (50% ligand density: 8.5 × 10^3^ ligands/NP; 10% ligand density: 1 × 10^3^ ligands/NP).

NPs were fabricated by using longer and shorter polymer chains. More specifically, the longer PEG_5k_-PLA_10k_ were functionalized with the peptide and the shorter ones (MeO-PEG_2k_-PLA_10k_) remained unfunctionalized. By decreasing the amount of longer functionalized polymer chains while increasing the amount of shorter polymer chains at the same time, it was possible to prepare NPs of varying surface ligand densities and to modulate NP size. Additionally, this approach allows the ligand to protrude from the particle surface and be more accessible for receptor interaction [32,33,34] because ligands have a higher flexibility when the distance between each ligand increases (Figure 2A) [21]. Ligand flexibility is of utmost importance to allow for binding of one particle to multiple receptors at the same time inducing Tie2 receptor clustering and finally, phosphorylation. Filling up the PEG shell with shorter-chain polymers is called backfilling and ensures NP shell integrity [21,34]. A PEG shell on the particle corona is essential for stealth properties such as the invisibility in biological systems thereby avoiding clearance of particles from the bloodstream [21,35]. Due to PEG’s high hydrophilic and flexible nature, a dense PEG shell protects NPs against opsonization, serum protein adsorption while the diffusivity of NPs is enhanced, and circulation time is prolonged [35,36]. The NP core was formed by PLGA. PLGA offers the possibility to make the NPs detectable by covalently attaching a fluorescent dye, and it gives particles sufficient stability to enhance structural integrity which allows the NP preparation by bulk nanoprecipitation [32]. Moreover, the lipophilic PLGA core additionally represents a potential reservoir for possible drug candidates which can be encapsulated for targeted drug delivery. Such particles would have a dual mode of action. All compounds are known for their excellent biocompatibility.

As expected, the NP size decreased with decreasing ligand density. With decreasing ligand density, the ligands experienced a space gain giving the ligand more flexibility in the PEG shell and the particle the opportunity to take smaller pack sizes. The resulting particles carried a negative zeta potential that came close to zero with increasing ligand content. Because the isoelectric point of the peptide is predicted to be at 10.5, the peptide will have a net charge of +1.0 at a physiological pH of 7.4 [37]. Therefore, it is obvious that the zeta potential was less negative with increasing numbers of positively charged ligand molecules on the particle surface. In any case, a negative zeta potential could be beneficial with respect to the trabecular outflow pathway as potential delivery route for NPs. Since the tissues of trabecular meshwork outflow pathway are negatively charged due to the presence of hyaluronic acid, small, negatively charged particles will most likely not be affected in their mobility and can freely diffuse through the trabecular meshwork [38,39,40]. The particles showed excellent stability in DPBS over 24 h. This guarantees NP integrity for optimal ligand display and a low risk for NP aggregation ensuring NP mobility as well. As it is a biologically demanding environment and an important parameter to know, the NP stability was further challenged in culture medium supplemented with 10% serum. Despite the stealth properties of a PEG shell, the NPs seemed to form a thin protein corona as a slight increase in particle size was observed. With regard to the environment to which the particles are exposed in the eye, the corona formation will most likely be much less pronounced, because the protein content in the aqueous humor equals to a serum concentration of only about 0.35% [41].

The primary HUVECs and the EA.hy926 cell line were included to evaluate the NP–cell interaction as well as the cellular response to NPs and compared to fibroblasts as control cell type. To estimate the cell response that can be expected for each cell type, the cells were evaluated for their Tie2 receptor expression levels. In accordance with Van Slyke et al., the cell line EA.hy926 expressed much higher levels of Tie2 compared to primary HUVECs [13]. Mostly low levels of Tie2 receptor were detected in fibroblasts which is consistent with observations from Teichert et al. [42]. With this knowledge, further experiments evaluating NP–cell interaction were performed.

In agreement with previous publications, NP–cell interaction depended largely on ligand density on the NP surface. In our case, the NP–cell interaction was improved with decreasing ligand density. This was counterintuitive because one would expect it vice versa. To explain this phenomenon, we had to take the size of NPs into account. In our study, the NP size increased with increasing ligand density. NPs with 0% ligand had a diameter of about 90 nm, while NPs with 100% ligand were about 120 nm in diameter. With the increasing ligand density, the NP–cell interaction decreased. To say it in other words: larger particles with higher ligand content were not taken up as efficiently as smaller particles with lower ligand content. This is well known from literature that small NPs (e.g., 50 nm) are internalized by cells better than large NPs (e.g., 100 nm) [43]. In our case, this allows for the assumption that NP size had a greater influence on NP–cell interaction than the ligand density. An issue of significant importance was that the interaction of NPs with fibroblasts was much lower compared to HUVECs and EA.hy926 cells. This in turn could have an advantage in terms of NP application. Let us take a look again at the trabecular outflow pathway as the potential delivery route. During glaucoma development, trabecular meshwork cells undergo a switch from a mesenchymal to a myofibroblast-like phenotype [2,44]. According to our results, these cells may eventually be expected to have a reduced NP interaction, which should enhance NP diffusion through the trabecular meshwork and allow NPs to reach their target cells in the SC.

In this study, we evaluated the cellular effects of Angpt-1 mimetic NPs by a Ca^2+^ mobilization assay as well as by a real time RT-PCR and Western blot analysis, regarding the expression of eNOS. Jho et al. demonstrated that Angpt-1 opposes VEGF-induced Ca^2+^ influx in endothelial cells in a dose-dependent manner [27]. Beside Angpt-1 mimetic NPs we evaluated a more stable variant called COMP Angpt-1 instead of recombinant Angpt-1, which forms pentamers rather than tetramers [11]. In contrast to COMP Angpt-1, Angpt-1 mimetic NPs at a concentration of 150 pM reversed the effect of VEGF and reduced the Ca^2+^ concentration dependent on the ligand density. In HUVECs, NPs with a ligand density of 25% opposed VEGF-induced Ca^2+^ influx significantly. The fact that cellular effects were more pronounced at lower ligand density may be related to the above described less intense NP–cell interaction at higher ligand densities. This also fits well to the results of Van Slyke et al. who demonstrated that too high concentrations of clustered Angpt-1 mimetic peptide were not capable of activating the Tie2 receptor [13]. A reason for the failure of COMP Angpt-1 in counteracting VEGF-induced Ca^2+^ influx in HUVECs, could also be related to the fact that COMP-Angpt-1 does not activate the Tie2 receptor in the same way as the native form does [45]. However, since the tone of vascular smooth muscle cells such as SC cells is controlled primarily by Ca^2+^ levels, reduced intracellular Ca^2+^ levels could therefore increase cell relaxation which should be beneficial for aqueous humor drainage in glaucoma [5,46].

One of the downstream targets of the Tie2 signaling cascade is eNOS [47]. For eNOS expression analysis, we used NPs with a ligand density ranging from 0–25% as they showed high NP–cell interaction. In our study, a trend of increasing eNOS mRNA in HUVECs was observed after both COMP Angpt-1 as well as Angpt-1 mimetic NP treatment with increasing concentration and ligand density, respectively. This could be beneficial for glaucoma therapy, as eNOS overexpressing mice have been demonstrated to have a decreased IOP and increased pressure-dependent outflow facility [48]. However, our results are not significant and at the protein level, this trend could not be confirmed. Regarding the effect of angiopoietins in the Tie2-AKT-eNOS pathway, an analysis of the different phosphorylation patterns would be more straightforward. Therefore, further studies are needed with regard to the phosphorylation state of Tie2, AKT and eNOS.

## 4. Materials and Methods

### 4.1. Materials

Hydroxyl poly(ethylene glycol)carboxylic acid with a molecular mass of 5000 g/mol (COOH-PEG5k-OH) was purchased from Jenkem Technology USA Inc. (Allen, TX, USA), hydroxyl poly(ethylene glycol) methyl ether with a molecular mass of 2000 g/mol (MeO-PEG2k-OH) and poly (lactide-co-glycolide) (PLGA), ester terminated as well as carboxy terminated, with a molecular mass of 13,400 g/mol (Resomer RG 502 and RG 502 H) were purchased from Sigma-Aldrich (Taufkirchen, Germany). Angiopoietin-1 (Angpt-1) mimetic peptide sequence containing a Leucine residue (LHHHRHSF) was acquired by GenScript Biotech (Leiden, The Netherlands). Fura-2 AM and CellMask Green Plasma Membrane Stain were both purchased from Fisher Scientific GmbH (Schwerte, Germany).

If not stated otherwise, all other chemicals were purchased from Sigma-Aldrich in analytical grade. Ultrapure water for dialysis and NP preparation was obtained from a Milli-Q water purification system (Millipore, Schwalbach, Germany).

### 4.2. Polymer Synthesis and Characterization

MeO-PEG_2k_-PLA_10k_ and COOH-PEG_5k_-PLA_10k_ block copolymers were synthesized via a ring-opening polymerization as described previously [33]. The molecular weight of the polymers was determined by NMR spectroscopic analysis in deuterated chloroform at 295 K using a Avance 400 spectrometer (Bruker BioSpin GmbH, Rheinstetten, Germany; Figure S2).

For polymer modification with Angpt-1 mimetic peptide sequence (LHHHRHSF), COOH-PEG_5k_-PLA_10k_ was covalently linked to the leucine residue of LHHHRHSF as previously described [20,33].

### 4.3. Peptide Quantification and Coupling Efficiency Determination

Resulting LHHHRHSF coupling efficiency for synthesized LHHHRHSF-PEG_5k_-PLA_10k_ was determined via independent measurement of the molar concentration of both PEG and LHHHRHSF. For LHHHRHSF quantification, a previously described variant of the Pauly reaction was applied [49]. For the determination of the PEG content a colorimetric iodine complexing assay was used. The procedure was as follows: LHHHRHSF modified polymer (PLA_10k_-PEG_5k_-CON-LHHHRHSF) was dissolved in acetonitrile (ACN; 10 mg/mL). A 400 µL measure of the polymer solution was added dropwise while vigorously stirring 0.1 × Dulbecco’s phosphate-buffered saline (DPBS; 4 mL) and kept stirring for 2 h at room temperature (RT) to remove the organic solvent. The resulting polymer micelle solution was concentrated via centrifugation at 1400× *g* for 30 min using Pall Microsep filters (molecular weight cut-off, 30 kDa; Pall Corporation, New York, NY, USA).

For the Pauly reaction, 75 µL of the concentrated micelle solution were mixed with 15 µL of 1% (*m*/*v*) sulfanilic acid dissolved in 1.4 N hydrochloric acid and incubated for 5 min on ice. Next, 15 µL of 5% (*m*/*v*) aqueous sodium nitrite solution were added. After three minutes of incubation, samples were taken from the ice and 150 µL of ethanol (70%; *v/v*) were added to each sample. A measure of 180 µL of the mixture was pipetted into a 96-well plate (Greiner Bio One, Frickenhausen, Germany) and absorbance was measured at λ = 490 nm using a FluoStar Omega fluorescence microplate reader (BMG Labtech, Ortenberg, Germany). Dilutions of LHHHRHSF (0–175 μg/mL) served as calibration.

The iodine complexing assay for PEG quantification was performed as previously described [50]. MeO-PEG_5k_ served as calibration. For the determination of the coupling efficiency, the molar LHHHRHSF content was finally normalized to the molar PEG content.

### 4.4. PLGA Labeling with Fluorescent Dye

Particles were made detectable by covalently linking a fluorescent dye (CF^TM^ 647) to the core-forming PLGA prior to NP preparation. In brief, CF^TM^ 647 amine (one equivalent) and carboxylic acid-terminated PLGA (Resomer RG 502H; one equivalent) were diluted in dimethylformamide (DMF). Twenty equivalents of (1H-Benzotriazole-1-yl)-1,1,3,3-tetramethyluronium hexafluorophosphate (HBTU) and forty equivalents of diisopropylethylamine (DIPEA) were added to the solution and allowed to react overnight at RT. Labeled PLGA was precipitated using diethyl ether, centrifuged, and diluted again in ACN. This procedure was repeated three times to remove unreacted reactants. Finally, fluorescently labeled PLGA was dried and stored at −80 °C until use.

### 4.5. NP Preparation

NPs were fabricated by nanoprecipitation using a standard solvent evaporation technique in accordance to previously published protocols [21,33]. In short, appropriate amounts of PEG-PLA polymers and PLGA were combined in a 70/30 (*m*/*m*) ratio and diluted in ACN to a final concentration of 10 mg/mL. For particles with different ligand contents (as indicated), functionalized (LHHHRHSF-PEG_5k_-PLA_10k_) was mixed with unfunctionalized (MeO-PEG_2k_-PLA_10k_) polymer at desired ratios, while keeping the above-mentioned molar ratio of PEG-PLA to PLGA constant. Finally, the polymer solution was added dropwise into vigorous stirring 0.1 × DPBS and kept stirring for 4 h under the fume hood at RT, allowing the organic solvent to evaporate.

Then, the obtained NP dispersions were concentrated by centrifugation at 1400× *g* for 30 min using Pall Microsep filters (molecular weight cut-off, 30 kDa). The molar concentration of NPs was determined and calculated as previously described considering the exact gravimetrical NP content, determined by lyophilization; the PEG content of NPs, determined by the iodine complexing assay (see below); the hydrodynamic diameter of the NPs determined by dynamic light scattering (DLS); and the density of NPs (1.25 g/cm^3^ [36]) [20,21]. Finally, the concentration of NP stock dispersion was adjusted to 3 nM and stored in the fridge (2–8 °C) until use.

### 4.6. NP Characterization

The hydrodynamic diameter (reported as size in the following) and the zeta potential of the NPs was determined by DLS and measuring the electrophoretic mobility, respectively, using a Malvern Zetasizer Nano ZS (Malvern, Herrenberg, Germany). For DLS and zeta potential measurements, particle stock solutions were diluted in DPBS (1:10) and the general-purpose mode with automatic measurement position was applied. For zeta potential measurements, particles were analyzed using capillary cells (Malvern, Herrenberg, Germany). To determine NP stability, NPs were incubated for 25 h at 37 °C in DPBS as well as Dulbecco’s modified Eagle’s medium (DMEM; Pan Biotech GmbH, Aidenbach, Germany) containing 10% (*v/v*) fetal bovine serum (FBS) and 0.01% (*m*/*v*) sodium azide (Merck KGaA, Darmstadt, Germany) for 24 h. At indicated time points, samples were taken, and the size was measured at 37 °C as described. Data were recorded using the Malvern Zetasizer software 7.11 (Malvern Instruments, Worcestershire, UK).

### 4.7. Calculation of the Number of Ligands Per Particle

The absolute number of ligands per particle for each particle formulation (100 to 0% ligand density) was calculated according to Abstiens et al. [20]. Therefore, a spherical NP shape was assumed, allowing us to calculate number of ligands per particles from ligands/µm^2^.

### 4.8. Cell Culture

HUVECs were obtained from PromoCell GmbH (Heidelberg, Germany) and were used until passage 6. HUVECs were cultured in EBM^TM^-2 basal medium supplemented with EGM^TM^-2 supplements and 2% (*v/v*) FBS, all from Lonza Group Ltd. (Basel, Switzerland). Primary cultures of human foreskin fibroblasts were cultured in DMEM supplemented with 4.5 g/L glucose and 10% (*v/v*) FBS (Thermo Fisher Scientific, Waltham, MA, USA). Vascular endothelial cell line (EA.hy926) was purchased from ATCC (Manassas, VA, USA) and cultured in DMEM containing 1.5 g/L sodium bicarbonate and 10% (*v/v*) FBS. For the Ca^2+^ mobilization assay, Leibovitz’s L-15 medium was purchased from Thermo Fisher Scientific (Gibco, NY, USA). If not otherwise stated, cells were serum starved overnight and incubated with either particles or COMP Angpt-1 (Enzo Life Sciences, Farmingdale, NY, USA) at indicated concentrations.

### 4.9. Confocal Laser Scanning Microscopy (CLSM) Analysis of Tie2 Receptor Expression

HUVECs, EA.hy926 cells and fibroblasts were seeded into Ibidi 8-well μ-slides (Ibidi GmbH, Planegg, Germany) at a density of 2.5 × 10^4^ cells/well and cultured for 24 h at 37 °C and 5% CO_2_. Thereafter, cells were washed with 0.1-fold phosphate buffer (0.1 × PBS) and fixed in 4% paraformaldehyde (PFA) for 10 min. After two washing steps with 0.1-fold PBS, cells were permeabilized with 0.5% Triton X-100 diluted in 0.1 × PBS for additional 10 min. After cells were blocked for 30 min with blocking buffer (2% bovine serum albumin (BSA) diluted in 0.1 × PBS and supplemented with 0.1% Triton X-100), cells were washed with dilution buffer (0.1% BSA diluted in 0.1 × PBS containing 0.1% Triton X-100) and incubated for one hour with 150 µL of primary mouse anti-human Tie2 monoclonal antibody (10 µg/mL; R&D-Systems, Inc., Minneapolis, MN, USA) diluted in dilution buffer. Afterwards cells were washed with 0.1 × PBS containing 0.1% BSA (washing buffer) followed by incubation with secondary Alexa fluor 488-labeled goat anti-mouse antibody diluted in dilution buffer (1:1000) for one hour at RT. Finally, cells were washed twice with washing buffer to remove excess antibody and embedded in Dako Fluorescence Mounting Medium (Agilent Technologies, Santa Clara, CA, USA). Control cells were stained with secondary antibody without having been previously incubated with the primary antibody. Cells were analyzed using a Zeiss LSM 510 Meta confocal microscope (Carl Zeiss Microscopy GmbH, Jena, Germany).

### 4.10. Flow Cytometry Analysis of Tie2 Receptor Expression

HUVECs, EA.hy926 cells and fibroblasts were trypsinized and seeded into centrifuge tubes at a density of 1 × 10^6^ cells/tube. The obtained cell suspension was washed with 0.1 × PBS and fixed in 4% PFA for 10 min. The following permeabilization and staining procedures were the same as for CLSM analysis except that the fixed cells were centrifuged (200× *g*; 5 min) after each incubation step. After cells were finally stained with the secondary antibody, they were resuspended in 0.1 × PBS and analyzed using a BD FACS Canto II (BD, Heidelberg, Germany). Again, control cells were incubated with secondary antibody without having been previously incubated with the primary antibody. Tie2-derived fluorescence was excited at 488 nm, and the emission was recorded using a 530/30 nm bandpass filter. The appropriate cell population was gated and analyzed using Flowing software 2.5.1 (Turku Centre for Biotechnology, Turku, Finland).

### 4.11. CLSM Analysis of NP–Cell Interaction

To obtain a first impression of NP–cell interaction HUVECs, EA.hy926 cells and fibroblasts were seeded into Ibidi 8-well μ-slides at a density of 5.0 × 10^4^ cells/well (HUVECs and EA.hy926 cells) or 2.5 × 10^4^ cells/well (fibroblasts) and cultured for 24 h at 37 °C and 5% CO_2_. Beforehand, cells were serum starved for 24 h. Freshly prepared NPs of varying ligand density (100% to 0%) with a CF647 modified PLGA core were diluted to 300, respectively, 15 pM in appropriate pre-warmed serum-free culture medium. Prior to NP treatment, cells were stained with CellMask green plasma membrane stain (1:400) for 30 min. Thereafter, cells were washed and 200 μL of NP dilutions were added in each well. Cells were incubated for two hours at 37 °C and 5% CO_2_. Subsequently, cells were washed with DPBS and fixed in 4% PFA in DPBS for 15 min at RT. Cells were washed again, nuclei were stained with 4′,6-diamidino-2-phenylindole (DAPI) and cells were finally embedded in Dako Fluorescence Mounting Medium after an additional washing procedure. Samples were examined using a Zeiss LSM 710.

### 4.12. Flow Cytometry Analysis of NP–Cell Interaction

To determine NP–cell interaction, HUVECs, EA.hy926 and fibroblasts were seeded at a density of 2 × 10^4^, 2 × 10^5^ and 1 × 10^5^ cells/well, respectively, and incubated for 94 h (HUVECs) or 24 h (EA.hy926 cells and fibroblasts) at 37 °C and 5% CO_2_. Subsequently, cells were serum starved for 24 h. Freshly prepared CF647-labeled NPs with indicated ligand densities were diluted to 300, respectively, 15 pM in appropriate culture medium. Cells were washed and incubated with NP dilutions for two hours at 37 °C. Afterwards, cells were washed with DPBS, trypsinized, and centrifuged for 5 min (200 g; 4 °C). The washing step was repeated twice. Final cell suspensions in DPBS were analyzed using a BD FACS Canto II. NP-associated fluorescence was excited at 633 nm, and the emission was measured using a 660/20 nm bandpass filter.

### 4.13. Ca^2+^ Mobilization Assay

To investigate NP binding to Tie2 receptor, intracellular Ca^2+^ levels were measured using fura-2 as a Ca^2+^ chelator as previously described, with slight modifications [33]. After HUVECs and EA.hy926 cells were trypsinized, collected and incubated in Leibovitz’s L-15 medium containing 5 μM fura-2 AM, 2.5 mM probenecid, and 0.05% Pluronics F-127 for 2 h at RT under gentle shaking (50 rpm). Subsequently, cells were collected by centrifugation and resuspended in Leibovitz’s L-15 medium at a concentration of 1.6 × 10^6^ cells/mL. The samples of 20 µL of NPs or COMP Angpt-1 at indicated concentrations were supplemented with or without VEGF (for a final concentration of 1 µg/mL; BioLegend San Diego, CA, USA), were pipetted into 96-well plates (half-area, costar, Corning, Inc., Kennebunk, ME, USA). Finally, 80 µL of cell suspension for a final cell concentration of 1.3 × 10^5^ cells/well was added and fluorescence signal was measured after 10 min using a FluoStar Omega fluorescence microplate reader with excitation filters at 340/20 nm and 380/20 nm and emission filter at 510/20 nm, respectively. To check if the cells had been successfully loaded with fura-2 AM, maximum and minimal ratio of Ca^2+^-bound to Ca^2+^-unbound fura-2 was measured by incubating loaded cells with 0.1% (*v/v*) Triton-X 100 respectively 0.1% (*v/v*) Triton-X 100 combined with 45 mM ethylene glycol-bis(2-aminoethylether)-N,N,N′,N′-tetraacetic acid (EGTA, data not shown).

### 4.14. Protein and RNA Isolation

After HUVECs were incubated with Angpt-1 mimetic NPs and COMP Angpt-1 for 24 h, as indicated, cells were washed twice with DPBS and lysed with 500 µL TRIzol^TM^ reagent (Invitrogen, Thermo Fisher Scientific, New York, NY, USA). Total RNA and protein were isolated according to the manufacturer’s instructions (TRIzol^TM^ reagent).

### 4.15. mRNA Isolation and Real-Time RT-PCR

To investigate the expression of eNOS on mRNA level, first-strand cDNA was prepared from total RNA using the iScript cDNA Synthesis Kit (BioRad, München, Germany) according to the manufacturer’s instructions. Real-time RT-PCR was performed on a CFX Connect Real-Time PCR Detection System (BioRad) with the temperature profile as follows: 50 cycles of 10 s melting at 95 °C and one minute of annealing and extension at 60 °C. All primer pairs were purchased from Invitrogen, extended over exon-intron boundaries and were as follows: 5′-CAGATGATCCCCCAGAACTC-3′ (human eNOS forward); 5′-CAGGGCTGCAAACCACTC-3′ (human eNOS reverse), 5′-GAAGTTCCTGGTCCACAACG-3′ (human RPL32 forward), 5′-GCGATCTCGGCACAGTAAG-3′ (human RPL32 reverse). RNA that was not reverse transcribed served as negative control for real-time RT-PCR.

### 4.16. Western Blot Analysis

To examine eNOS expression, proteins of HUVECs were dissolved in 1% SDS containing protease (1:100; Merck, Darmstadt, Germany) and phosphatase inhibitors (1:100; Roth, Karlsruhe, Germany). Protein content was measured with the bicinchoninic acid assay (ROTI Quant universal, Karlsruhe, Germany) using the NanoDrop-1000 device (Peqlab Biotechnologie GmbH, Erlangen, Germany). Western blot analysis was performed with specific antibodies as described previously [51]. Briefly, 2.5 µg of each sample was denatured by boiling for 5 min. Proteins were separated by SDS-polyacrylamide gel electrophoresis (PAGE) and transferred to polyvinylidene fluoride (PVDF) membranes (Merck, Darmstadt, Germany) overnight by tank blot (17 h, 30 V, 60 mA; PeqLab Biotechnologie GmbH, Erlangen, Germany). Membranes were blocked in a 5% blocking solution of BSA for one hour at room temperature. Specific antibodies were used as follows: rabbit anti-eNOS (1:500; 9572S, Cell signaling, Cambridge UK), rabbit anti-α-tubulin (1:2500, 600-401-880, Rockland Immunochemicals Inc., Gilbertsville, PA, USA), goat anti-rabbit horseradish peroxidase (HRP) (1:2000, 7074S, Cell signaling, Cambridge UK). Chemiluminescence was detected on a LAS 3000 imaging workstation (Fujifilm, Düsseldorf, Germany). For normalization of the signal intensity, α-tubulin was used as a loading control. The intensity of the bands detected by Western blotting was determined using ImageLab software (Bio-Rad, Feldkirchen, Germany).

### 4.17. Statistical Analysis

All data are presented as means ± standard deviation (SD) or standard error of the mean (SEM), of at least 3 measurements. Standard deviations of relative values were calculated according to the rules of error propagation (Figure 1B, Figure 3B and Figure 4B–D). One-way ANOVA, followed by Tukey’s multiple comparisons test (Figure 3B, Figure 5 and Figure 6A,C) as well as a two-way ANOVA and Tukey’s multiple comparisons test (Figure 4C) were performed using GraphPad Prism 8.3.0 (GraphPad Software Inc., La Jolla, CA, USA) to assess statistical significance. Statistical significances were assigned as indicated.

## 5. Conclusions and Outlook

The SC forms the central outflow pathway for aqueous humor and is thus a key player in the pathogenesis of POAG. This study demonstrated Angpt-1 mimetic NPs as a promising tool to target SC cells and to restore SC cell function for glaucoma therapy. A major goal for further studies will be to enhance the specific interaction of NPs with cells. Because the Angpt-1 sequence is positively charged it may interact with the negatively charged particle core. This may provoke the Angpt-1 mimetic peptide sequence to flip towards the particle surface and no longer be available on the NP surface for specific NP–Tie2 interaction.

However, in the long run, Angpt-1 mimetic NPs potentially offer the possibility to have a dual mode of action. First, when Angpt-1 NPs bind to the Tie2 receptor, the impaired intracellular signaling cascade will be reactivated. Second, by encapsulating a potential anti-glaucoma drug into the NP core, it will target another signaling cascade. Such a delivery system could be extremely potent for glaucoma therapy as it offers the possibility to address two different mechanisms involved in glaucoma progression. Therefore, the development of Angpt-1 mimetic NPs is an initial step in the right direction to restore the impaired SC cells essential for long-lasting IOP reduction.

## Data Availability

Date is contained in the article and supplementary materials.

## Supplementary Materials

The following are available online at https://www.mdpi.com/article/10.3390/ph15010018/s1, Figure S1: NPs are well tolerated by HUVECs, EA.hy926 and fibroblasts, Figure S2: H-NMR (CDCl_3_, 400 MHz) spectra of MeO-PEG_2k_-PLA_10k_ (A) and COOH-PEG_5k_-PLA_10k_ (B): 1.55 ppm (-C(CH_3_)H-), 3.43 ppm (H_3_COCH_2_CH_2_-), 3.64 ppm (-OCH_2_CH_2_-), 5.17 ppm (-C(CH_3_)H-), 7,26 ppm (solvent peak).
