# Supplementary material for: Angiopoietin-1 Mimetic Nanoparticles for Restoring the Function of Endothelial Cells as Potential Therapeutic for Glaucoma
Source: Pharmaceuticals (Basel). 2021 Dec 24;15(1):18. doi: 10.3390/ph15010018 (PMC8780450; doi:10.3390/ph15010018)
Supplement: Supplementary file 1 [file pharmaceuticals-15-00018-s001.zip › pharmaceuticals-1499889-supplementary.pdf]

pharmaceuticals

Supplementary Materials: Angiopoietin-1 Mimetic Nanoparticles for Restoring the Function of Endothelial Cells as Potential Therapeutic for Glaucoma

Raphael Mietzner, Ramona Pawlak, Ernst R. Tamm, Achim Goepferich, Rudolf Fuchshofer and Miriam Breunig*

MTT (3-(4,5-dimethylthiazol-2-yl)-2,5-diphenyltetrazolium bromide)-assay was used to determine the viability of HUVECs, EA.Hy926 cells and fibroblasts treated with NPs containing a ligand density of 100% at indicated concentrations. The assay was performed according to previous publication with slight modifications [1]. Cells were seeded in a 96-well plate at a density of 12,000 cells/well for EA.hy926 cells, 10,000 cells/well for HUVECs and 7,500 cells/well for fibroblasts. Cells were cultivated for 24 hours at 37 °C and 5% CO_2. Subsequently, cells were washed with DPBS and the culture medium was replaced by 100 µL NP dilutions ranging from 0.01 to 0.4 mg/mL in appropriate culture medium supplemented with 0.35% FBS. After 4 hours of incubation with NPs, NP containing supernatant was replaced by fresh culture medium containing 10% FBS and cells were incubated for additional 19 hours at 37 °C and 5% CO_2. Thereafter, culture medium was replaced by 200 µL of MTT (625 µg/mL in serum-free culture medium) and further incubated for 5 hours at 37 °C. Then, the supernatant was replaced by 60 µL DPBS containing 10% (w/v) sodium dodecyl sulfate (SDS). The cells were incubated overnight in the dark and room temperature. The absorbance of formazan was measured at 570-690 nm using a microplate reader (Fluostar Omega; BMG Labtech GmbH, Ortenberg, Germany). As positive and negative control served cells treated with DPBS and 0.1% (w/v) SDS, respectively. The results were calculated as the mean percentage of viability in relation to the positive control. Viabilities below 70% were considered as cytotoxic. (n=6).

Figure S1 shows the cell viability data of NP-treated HUVECs, EA.hy926 and fibroblasts. Data indicate that all analyzed cell types tolerated well all used NP concentrations.

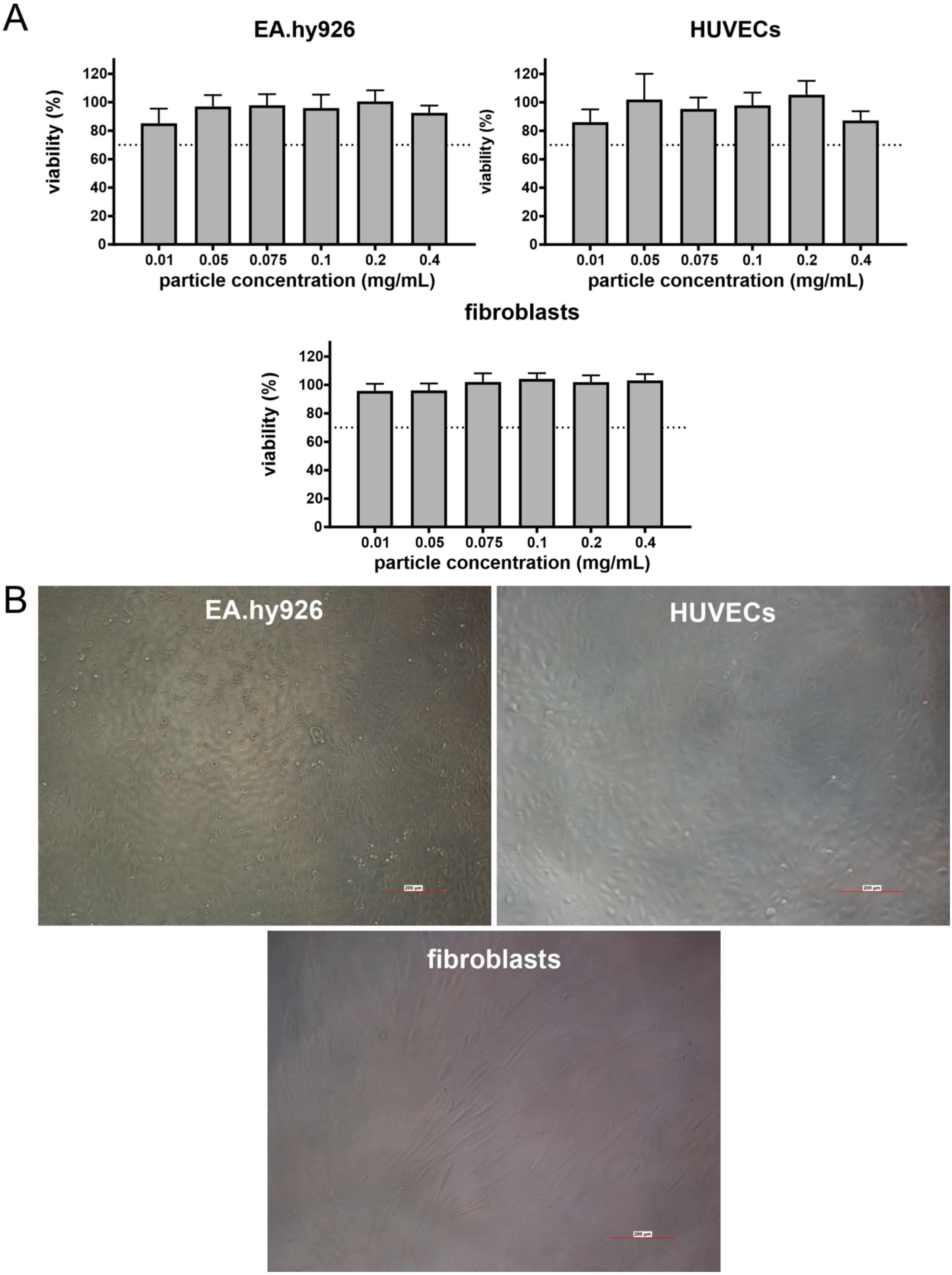
A
EA.hy926
HUVECs
fibroblasts
viability (%)
0
20
40
60
80
100
120
0.01
0.05
0.075
0.1
0.2
0.4
particle concentration (mg/mL)
B
EA.hy926
HUVECs
fibroblasts

Figure S1. NPs are well tolerated by HUVECs, EA.hy926 and fibroblasts. (A) Cells were incubated for 4 hours with different NP concentrations ranging from 0.01 to 0.4 mg/mL in appropriate culture medium containing 0.35% FBS. Data are presented as mean percentage of viability ± SD of the mean (n=6). (B) Representative brightfield images of cells treated with NPs (0.4 mg/mL) before MTT reagent was added. Bars indicate 200 µm.

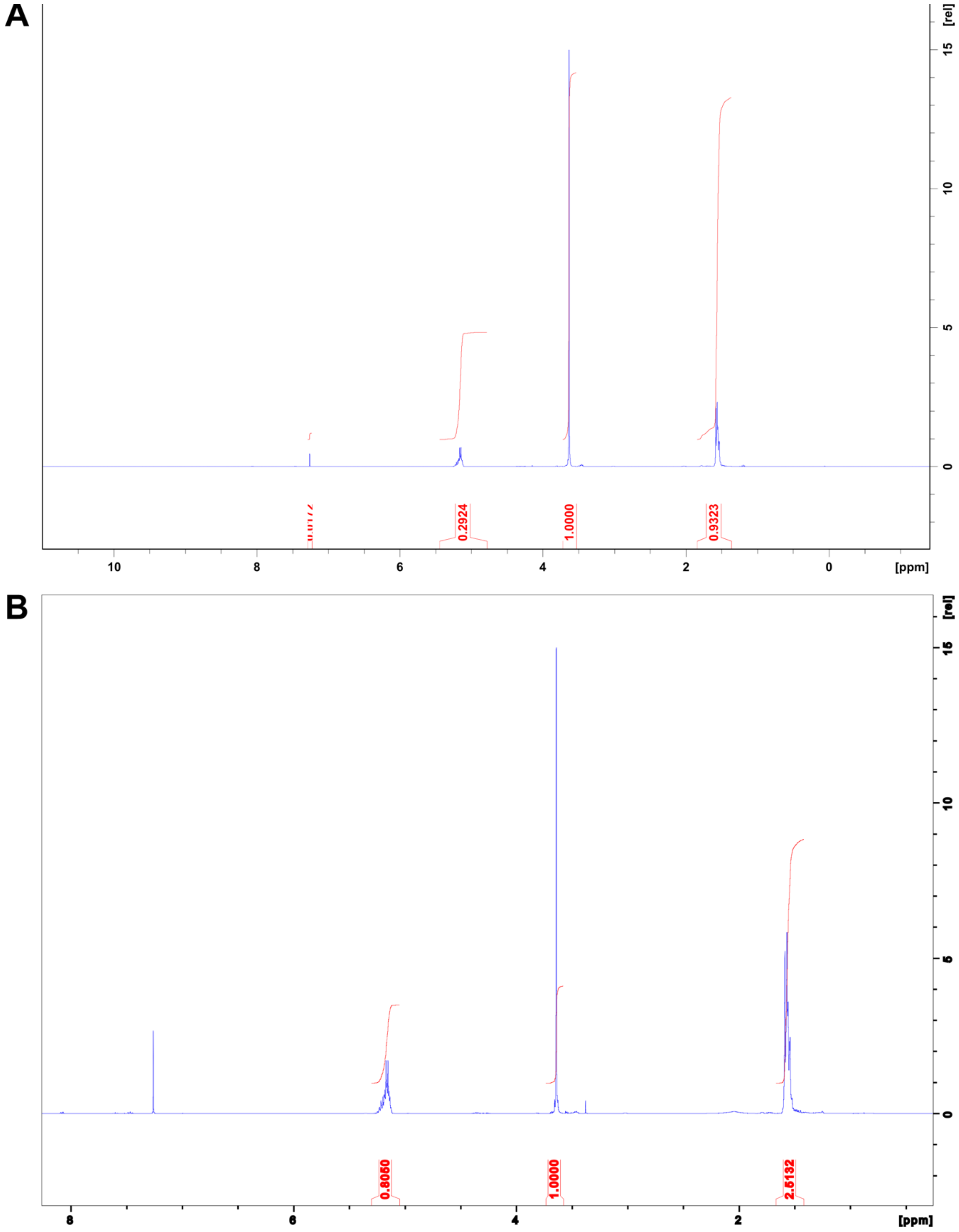

Figure S2. H-NMR ($CDCl_3$, 400 MHz) spectra of MeO-PEG$_{2k}$-PLA$_{10k}$ (A) and COOH-PEG$_{5k}$-PLA$_{10k}$ (B): 1.55 ppm (-C(CH_3)H-),3.43 ppm ($H_3COCH_2CH_2$-) 3.64 ppm (-OCH_2CH_2-), 5.17 ppm (-C(CH_3)H-), 7,26 ppm (solvent peak).

References

1.Mietzner, R.; Kade, C.; Froemel, F.; Pauly, D.; Stamer, W.D.; Ohlmann, A.; Wegener, J.; Fuchshofer, R.; Breunig, M. Fasudil Loaded PLGA Microspheres as Potential Intravitreal Depot Formulation for Glaucoma Therapy. Pharmaceutics 2020, 12, doi:10.3390/pharmaceutics12080706.
